# Supplementary material for: Natural history of long-COVID in a nationwide, population cohort study
Source: Nat Commun. 2023 Jun 13;14:3504. doi: 10.1038/s41467-023-39193-y (PMC10263377; doi:10.1038/s41467-023-39193-y)
Supplement: Supplementary file 1 — Supplementary Information [file 41467_2023_39193_MOESM1_ESM.pdf]

Supplementary Figure 1. Self-completed Questionnaire

Q1. Have you ever had a positive Covid-19 test? (yes/no)

Q2. Have you had a Covid-19 vaccine? (yes/no)

Q3. What Covid-19 symptoms did you have? [only if Q1 yes] (select all that apply)
Fever; Cough; Change in taste; Change in smell; Tiredness (fatigue); Headache; Ear pain; Runny nose; Muscle aches or weakness; Joint pain; Breathlessness; Chest pain; Sore throat; Hoarse voice; Loss of appetite; Stomach (abdominal) pain; Diarrhoea; Confusion; Seizures; Hair loss; Unconscious/semi-conscious; I didn't have any symptoms [go to Q5]

Q4. How long did your symptoms last?
Less than 1 week;1-4 weeks; More than 4 weeks

Q5. How do you feel now?
Fully recovered; Partly recovered; Not recovered

Q6. Before your Covid-19 test, did you have any health issues or medical conditions?
Yes; No [go to Q8]

Q7. Which health issues or medical conditions did you have? (select all that apply)
Arthritis; Asthma/bronchitis/COPD; Cancer; Cystic fibrosis; Deep vein thrombosis; Depression/anxiety; Diabetes; Heart disease; Heart failure; High blood pressure; HIV; Home oxygen; Kidney disease; Liver disease; Neurological condition; Overweight; Obese; Pulmonary embolism; Pulmonary fibrosis; Stroke; I didn't have any of these [go to Q8]

Q8. Have you experienced any of the following in the last week? (select all that apply)
Dry cough; Cough with phlegm; Change in taste; Change in smell; Problems hearing; Problems with eyesight; Tired; Headache; Muscle aches/weakness; Pins and needles; Joint pain; Breathless; Chest pain; Palpitations; Poor appetite; Stomach (abdominal) pain; Feeling sick/vomiting; Diarrhoea; Constipation; Weight loss; Anxious/depressed; Confusion/difficulty concentrating; Dizzy/blackouts/fits; Balance problems; Skin rash; Problems sleeping; I haven't experienced any of these [go to Q10]

Q9. Are any of these new or worse since your Covid-19 test? (select all that apply)
Response list from Q8; None of these are new or worse [go to Q10]

Q10. Is your current health causing you difficulty with any of the following? Select all that apply.
Walking/getting around; Housework/DIY/chores; Working/studying; Washing/dressing; Exercise/sports; Hobbies; Relationships; None of these apply [go to Q12; if also no to Q2 go to Q13]

Q11. Which of the difficulties you're experiencing are new/worse since having your Covid-19 test? (select all that apply)
Response list from Q10; None of these are new or worse [go to Q13]

Q12. Has your job changed since your Covid-19 test?
No change; Different due to my Covid-19 illness; Different for other reasons; None of these apply

Q13. What is your ethnicity?
White Scottish; White Other British; White Irish; White Gypsy/Traveller; White Polish; Other white ethnic group; Mixed or multiple ethnic groups; Pakistani, Pakistani Scottish or Pakistani British; Indian, Indian Scottish or Indian British; Bangladeshi, Bangladeshi Scottish or Bangladeshi British; Chinese, Chinese Scottish or Chinese British; Other Asian, Asian Scottish or Asian British; African, African Scottish or African British; Other African; Caribbean, Caribbean Scottish or Caribbean British; Black, Black Scottish or Black British; Other Caribbean or Black; Arab, Arab Scottish or Arab British; Other ethnic group; Prefer not to say

Q14. How would you rate your health today?
EQ-5D thermometer (0 Worst health I can imagine – 100 Best health I can imagine)

Supplementary Figure 2. Stacked bar charts of symptoms at a. 12-month follow-up and b. 18-month follow-up

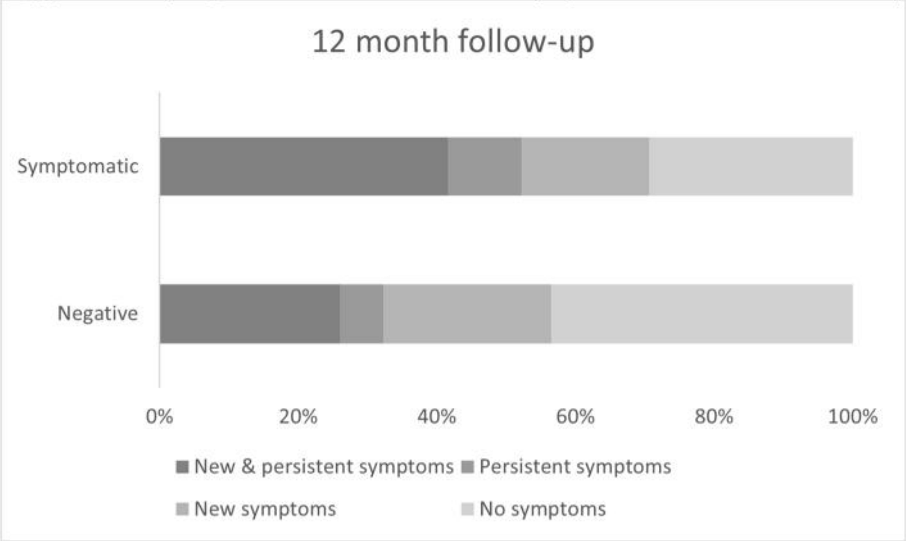

a.

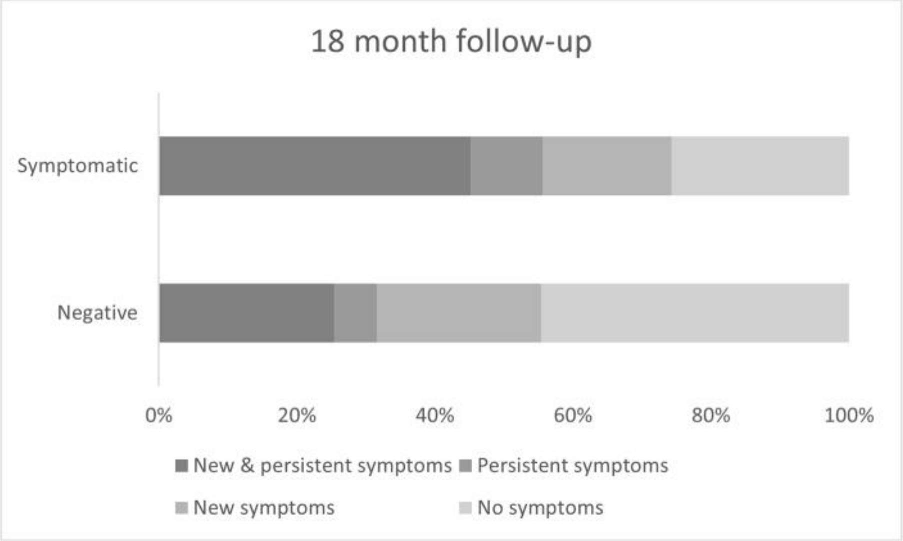

b.

New symptoms were not reported at 6-month follow-up, whereas persistent symptoms were reported at 6-month follow-up.

Supplementary Table 1. Characteristics of participants by recovery status trajectory between 6 and 18 months following symptomatic SARS-CoV-2 infection

	**Constant N=2,998**	**Deteriorated N=587**	**Improved N=611**	**P value**
	Med (IQR)	Med (IQR)	Med (IQR)	
**Age(years)**	51 (39-60)	52 (41-59)	52 (40-60)	0.419
**Sex**	N (%)	N (%)	N (%)	
Female	1,890 (63.0)	385 (65.6)	436 (71.4)	
Male	1,108 (37.0)	202 (34.4)	175 (28.6)	<0.001
**SIMD**				
1 (most deprived)	653 (21.8)	153 (26.1)	138 (22.6)	
2	623 (20.8)	148 (25.2)	129 (21.1)	
3	560 (18.7)	89 (15.2)	122 (20.0)	
4	590 (19.7)	113 (19.3)	110 (18.0)	
5 (least deprived)	572 (19.1)	84 (14.3)	112 (18.3)	0.012
**Ethnic group**				
White	2,816 (93.9)	563 (95.9)	583 (95.4)	
South Asian	40 (1.33)	Not disclosed	Not disclosed	
Black	13 (0.43)	Not disclosed	Not disclosed	
Other	39 (1.30)	Not disclosed	Not disclosed	
Missing	90 (3.00)	13 (2.21)	19 (3.11)	0.209
**Number of pre-existing health conditions**				
0	2,018 (67.3)	375 (63.9)	398 (65.1)	
1	378 (12.6)	81 (13.8)	89 (14.6)	
2-3	487 (16.2)	103 (17.6)	95 (15.6)	
≥4	115 (3.84)	28 (4.77)	29 (4.75)	0.494
**Pre-existing health conditions**				
Arthritis	235 (7.84)	62 (10.6)	62 (10.2)	0.031
Asthma/bronchitis/COPD	674 (22.5)	150 (25.6)	158 (25.9)	0.082
Cancer	30 (1.00)	Not disclosed	10 (1.64)	0.277
CHD	112 (3.74)	26 (4.43)	22 (3.60)	0.694
Cystic fibrosis	Not disclosed	Not disclosed	Not disclosed	0.046
Deep vein thrombosis	Not disclosed	Not disclosed	Not disclosed	0.245
Depression/anxiety	1,349 (45.0)	330 (56.2)	301 (49.3)	<0.001
Diabetes	169 (5.64)	37 (6.30)	39 (6.38)	0.677
High blood pressure	338 (11.3)	78 (13.3)	72 (11.8)	0.377
HIV	Not disclosed	Not disclosed	Not disclosed	0.543
Home oxygen	Not disclosed	Not disclosed	Not disclosed	0.321
Kidney disease	26 (0.87)	Not disclosed	Not disclosed	0.286
Liver disease	Not disclosed	Not disclosed	Not disclosed	0.934
Neurological condition	70 (2.33)	12 (2.04)	Not disclosed	0.028
Overweight	325 (10.8)	70 (11.9)	79 (12.90)	0.289
Obese	131 (4.37)	14 (2.39)	22 (3.60)	0.070
Pulmonary embolism	15 (0.50)	Not disclosed	Not disclosed	0.768
Pulmonary fibrosis	Not disclosed	Not disclosed	Not disclosed	0.670
Stroke	20 (0.67)	Not disclosed	Not disclosed	0.101
**Vaccinated**				
No	2,843 (94.8)	558 (95.1)	587 (96.1)	
1 dose	147 (4.90)	23 (3.92)	23 (3.76)	
≥2 doses	Not disclosed	Not disclosed	Not disclosed	0.029
**Variant period**				
Pre VOC	1,035 (34.5)	217 (37.0)	228 (37.3)	
No dominant (1)	1,475 (49.2)	287 (48.9)	288 (47.1)	
Alpha	401 (13.4)	69 (11.8)	76 (12.4)	
No dominant (2)	87 (2.90)	14 (2.39)	19 (3.11)	0.689

Kruskal Wallis test for continuous variables, Chi^2 test for categorical variables. All statistical tests are two-sided.

IQR inter-quartile range; N number; SIMD Scottish Index of Multiple Deprivation; COPD chronic obstructive pulmonary disease; CHD coronary heart disease; HIV human immunodeficiency virus; VOC variant of concern

Supplementary Table 2. Binary logistic regression of factors associated with improvement and deterioration in recovery status between 6 and 12 months (unadjusted odds ratios)

		Improvement (excluding those fully recovered at 6 months)		Deterioration (excluding those with no recovery at 6 months)	
		Referent no change N=6,082	Referent no change plus deterioration N=6,540	Referent no change N=10,877	Referent no change plus improvement N=12,056
		OR (95% CI)	OR (95% CI)	OR (95% CI)	OR (95% CI)
Age		0.99 (0.99,1.00)	0.99 (0.99,1.00)	0.99 (0.99,1.00)	0.99 (0.99,1.00)
Sex	Female	1	1	1	1
	Male	1.04 (0.92,1.17)	1.04 (0.92,1.17)	0.82 (0.73,0.92)	0.85 (0.75,0.95)
Ethnic group	White	1	1	1	1
	South Asian	0.93 (0.45,1.90)	0.93 (0.46,1.89)	1.17 (0.72,1.89)	1.22 (0.76,1.98)
	Black	2.17 (0.81,5.83)	2.39 (0.89,6.43)	0.78 (0.28,2.21)	0.74 (0.26,2.08)
	Other	1.51 (0.86,2.65)	1.54 (0.88,2.67)	0.82 (0.47,1.43)	0.81 (0.47,1.42)
	Missing	0.92 (0.64,1.33)	0.95 (0.66,1.37)	0.96 (0.67,1.37)	0.97 (0.68,1.38)
SIMD quintile	1 (most deprived)	1	1	1	1
	2	1.17 (0.98,1.38)	1.19 (1.00,1.41)	0.91 (0.77,1.07)	0.90 (0.77,1.05)
	3	1.32 (1.11,1.58)	1.36 (1.14,1.63)	0.75 (0.63,0.89)	0.73 (0.62,0.87)
	4	1.31 (1.10,1.57)	1.34 (1.12,1.60)	0.83 (0.70,0.98)	0.82 (0.70,0.97)
	5 (least deprived)	1.45 (1.20,1.73)	1.52 (1.27,1.82)	0.65 (0.55,0.78)	0.66 (0.55,0.78)
Pre-existing long-term conditions	0	1	1	1	1
	1	0.97 (0.83,1.14)	0.98 (0.84,1.15)	1.04 (0.88,1.22)	1.02 (0.87,1.20)
	2-3	0.83 (0.71,0.97)	0.83 (0.71,0.97)	1.12 (0.96,1.31)	1.11 (0.95,1.29)
	≥4	0.85 (0.65,1.12)	0.80 (0.61,1.05)	1.68 (1.31,2.17)	1.68 (1.31,2.16)
Asthma/bronchitis/ COPD	No	1	1	1	1
	Yes	0.88 (0.77,0.99)	0.86 (0.76,0.98)	1.21 (1.06,1.37)	1.20 (1.06,1.36)
CHD	No	1	1	1	1
	Yes	1.05 (0.79,1.41)	1.03 (0.77,1.36)	1.16 (0.88,1.53)	1.16 (0.88,1.52)
Depression/anxiety	No	1	1	1	1
	Yes	0.77 (0.68,0.86)	0.74 (0.66,0.83)	1.46 (1.31,1.63)	1.44 (1.29,1.61)
Diabetes	No	1	1	1	1
	Yes	0.92 (0.73,1.18)	0.92 (0.72,1.17)	1.17 (0.93,1.48)	1.17 (0.93,1.46)
Variant period	preVOC	1	1	1	1
	No dominant (1)	1.15 (0.99,1.33)	1.12 (0.97,1.30)	1.16 (1.01,1.34)	1.14 (0.99,1.32)
	Alpha	1.20 (0.93,1.57)	1.23 (0.95,1.59)	0.96 (0.74,1.26)	0.94 (0.72,1.23)
	No dominant (2)	0.70 (0.44,1.11)	0.69 (0.44,1.09)	0.95 (0.66,1.37)	0.99 (0.69,1.42)
	Delta	1.23 (1.06,1.42)	1.21 (1.05,1.40)	0.92 (0.80,1.06)	0.93 (0.81,1.07)

	No dominant (3)	0.52 (0.15,1.76)	0.53 (0.16,1.81)	0.41 (0.10,1.72)	0.43 (0.10,1.81)
Vaccinated at test	No	1	1	1	1
	1 dose	0.96 (0.75,1.23)	0.96 (0.75,1.23)	0.99 (0.79,1.24)	1.00 (0.80,1.25)
	≥2 doses	1.03 (0.90,1.18)	1.03 (0.90,1.18)	0.86 (0.76,0.97)	0.89 (0.78,1.00)
Infection severity	Not hospitalised	1	1	1	1
	Hospitalised	0.72 (0.57,0.91)	0.71 (0.56,0.89)	1.17 (0.90,1.51)	1.16 (0.90,1.49)

OR odds ratio; CI confidence interval; SIMD Scottish Index of Multiple Deprivation; COPD chronic obstructive pulmonary disease; CHD coronary heart disease; VOC variant of concern. All statistical tests are two-sided.

Supplementary Table 3. Binary logistic regression of factors associated with improvement and deterioration in recovery status between 6 and 18 months

		Improvement (excluding those fully recovered at 6 months)		Deterioration (excluding those with no recovery at 6 months)	
		Referent no change	Referent no change plus deterioration	Referent no change	Referent no change plus improvement
		N=2,102<br>OR (95% CI)	N=2,291<br>OR (95% CI)	N=3,402<br>OR (95% CI)	N=3,823<br>OR (95% CI)
Age		1.01 (0.99,1.01)	1.01 (0.99,1.01)	0.99 (0.99,1.01)	0.99 (0.99,1.01)
Sex	Female	1.00	1.00	1.00	1.00
	Male	0.80 (0.64,0.99)	0.78 (0.63,0.97)	0.98 (0.80,1.20)	1.01 (0.83,1.23)
Ethnic group	White	1.00	1.00	1.00	1.00
	South Asian	1.27 (0.46,3.52)	1.28 (0.47,3.49)	0.68 (0.26,1.75)	0.71 (0.28,1.83)
	Black	-	-	-	-
	Other	0.61 (0.17,2.21)	0.64 (0.18,2.27)	0.82 (0.34,1.97)	0.87 (0.36,2.08)
	Missing	1.06 (0.61,1.85)	1.18 (0.68,2.04)	0.75 (0.41,1.36)	0.75 (0.41,1.35)
SIMD quintile	1 (most deprived)	1.00	1.00	1.00	1.00
	2	1.01 (0.76,1.35)	0.98 (0.74,1.30)	1.07 (0.83,1.39)	1.08 (0.84,1.39)
	3	1.15 (0.86,1.54)	1.17 (0.88,1.57)	0.69 (0.52,0.92)	0.68 (0.51,0.91)
	4	1.02 (0.76,1.38)	1.04 (0.77,1.40)	0.87 (0.66,1.14)	0.87 (0.67,1.14)
	5 (least deprived)	1.24 (0.92,1.69)	1.29 (0.96,1.74)	0.67 (0.50,0.89)	0.66 (0.49,0.88)
Pre-existing long-term conditions	0	1.00	1.00	1.00	1.00
	1	1.12 (0.83,1.50)	1.06 (0.79,1.41)	1.03 (0.78,1.36)	1.00 (0.76,1.33)
	2-3	0.67 (0.50,0.90)	0.68 (0.51,0.90)	0.96 (0.73,1.27)	0.99 (0.75,1.30)
	≥4	0.73 (0.43,1.24)	0.75 (0.45,1.27)	1.06 (0.62,1.79)	1.02 (0.60,1.71)
Asthma/bronchitis/ COPD	No	1.00	1.00	1.00	1.00
	Yes	1.06 (0.84,1.34)	1.06 (0.84,1.33)	1.12 (0.90,1.40)	1.11 (0.89,1.39)
CHD	No	1.00	1.00	1.00	1.00
	Yes	0.89 (0.52,1.51)	0.89 (0.53,1.49)	1.17 (0.72,1.89)	1.20 (0.75,1.94)
Depression/anxiety	No	1.00	1.00	1.00	1.00
	Yes	0.75 (0.61,0.92)	0.72 (0.59,0.88)	1.56 (1.29,1.89)	1.58 (1.31,1.91)
Diabetes	No	1.00	1.00	1.00	1.00
	Yes	1.37 (0.88,2.14)	1.34 (0.87,2.07)	1.01 (0.66,1.55)	0.99 (0.65,1.51)
Variant period	preVOC	1.00	1.00	1.00	1.00
	No dominant (1)	0.98 (0.80,1.21)	0.98 (0.80,1.21)	0.92 (0.76,1.12)	0.93 (0.77,1.13)

	Alpha	1.07 (0.77,1.48)	1.11 (0.80,1.52)	0.81 (0.59,1.11)	0.81 (0.59,1.10)
	No dominant (2)	1.49 (0.77,2.87)	1.51 (0.79,2.89)	0.63 (0.32,1.25)	0.60 (0.30,1.18)
Vaccinated	No	1.00	1.00	1.00	1.00
	1 dose	0.63 (0.36,1.09)	0.65 (0.38,1.11)	0.86 (0.52,1.42)	0.90 (0.54,1.48)
	≥2 doses	0.35 (0.04,3.20)	0.40 (0.04,3.68)	3.96 (1.23,12.7)	4.79 (1.49,15.4)
Infection severity	Not hospitalised	1.00	1.00	1.00	1.00
	Hospitalised	0.74 (0.53,1.04)	0.74 (0.53,1.03)	1.17 (0.82,1.66)	1.15 (0.81,1.63)

OR odds ratio; CI confidence interval; SIMD Scottish Index of Multiple Deprivation; COPD chronic obstructive pulmonary disease; CHD coronary heart disease; VOC variant of concern. All statistical tests are two-sided.
Odds ratios are adjusted.

Supplementary Table 4. Binary logistic regression of factors associated with improvement and deterioration in recovery status between 6 and 18 months (unadjusted odds ratios)

		Improvement (excluding those fully recovered at 6 months)		Deterioration (excluding those with no recovery at 6 months)	
		Referent no change N=2,102	Referent no change plus deterioration N=2,291	Referent no change N=3,402	Referent no change plus improvement N=3,823
		OR (95% CI)	OR (95% CI)	OR (95% CI)	OR (95% CI)
Age		1.00 (0.99,1.01)	1.00 (0.99,1.01)	1.00 (0.99,1.01)	1.00 (0.99,1.01)
Sex	Female	1	1	1	1
	Male	0.85 (0.69,1.05)	0.84 (0.69,1.03)	0.89 (0.74,1.08)	0.92 (0.77,1.11)
Ethnic group	White	1	1	1	1
	South Asian	1.33 (0.49,3.61)	1.38 (0.52,3.69)	0.59 (0.23,1.50)	0.63 (0.25,1.60)
	Black	-	-	-	-
	Other	0.56 (0.16,1.98)	0.59 (0.17,2.06)	0.72 (0.31,1.72)	0.78 (0.33,1.83)
	Missing	1.03 (0.60,1.78)	1.14 (0.66,1.96)	0.74 (0.41,1.33)	0.74 (0.41,1.34)
SIMD quintile	1 (most deprived)	1	1	1	1
	2	1.03 (0.78,1.36)	0.99 (0.76,1.31)	1.04 (0.81,1.34)	1.05 (0.82,1.35)
	3	1.20 (0.90,1.61)	1.23 (0.92,1.63)	0.67 (0.50,0.89)	0.66 (0.50,0.88)
	4	1.09 (0.81,1.47)	1.11 (0.83,1.49)	0.80 (0.61,1.04)	0.80 (0.61,1.05)
	5 (least deprived)	1.35 (1.00,1.82)	1.39 (1.04,1.87)	0.61 (0.45,0.81)	0.60 (0.45,0.80)
Pre-existing long-term conditions	0	1	1	1	1
	1	1.09 (0.82,1.43)	1.03 (0.78,1.35)	1.15 (0.88,1.50)	1.13 (0.87,1.47)
	2-3	0.66 (0.51,0.86)	0.66 (0.51,0.85)	1.20 (0.94,1.53)	1.22 (0.96,1.55)
	≥4	0.74 (0.48,1.15)	0.74 (0.48,1.13)	1.48 (0.96,2.29)	1.45 (0.95,2.22)
Asthma/bronchitis/ COPD	No	1	1	1	1
	Yes	0.92 (0.74,1.14)	0.91 (0.74,1.13)	1.24 (1.01,1.53)	1.23 (1.01,1.51)
CHD	No	1	1	1	1
	Yes	0.75 (0.46,1.22)	0.74 (0.46,1.19)	1.28 (0.82,1.99)	1.30 (0.84,2.01)
Depression/anxiety	No	1	1	1	1
	Yes	0.73 (0.60,0.88)	0.70 (0.58,0.84)	1.66 (1.39,1.99)	1.67 (1.40,2.00)
Diabetes	No	1	1	1	1
	Yes	0.97 (0.66,1.43)	0.96 (0.66,1.39)	1.17 (0.81,1.69)	1.15 (0.80,1.66)
Variant period	preVOC	1	1	1	1
	No dominant (1)	0.93 (0.76,1.15)	0.93 (0.76,1.14)	0.93 (0.76,1.13)	0.95 (0.78,1.15)
	Alpha	0.95 (0.70,1.30)	0.99 (0.74,1.35)	0.81 (0.60,1.09)	0.82 (0.61,1.10)
	No dominant (2)	1.09 (0.62,1.93)	1.16 (0.66,2.02)	0.73 (0.41,1.31)	0.71 (0.40,1.26)

Vaccinated at test	No	1	1	1	1
	1 dose	0.73 (0.45,1.17)	0.75 (0.47,1.21)	0.78 (0.50,1.22)	0.79 (0.50,1.23)
	≥2 doses	0.48 (0.06,4.13)	0.54 (0.06,4.66)	3.60 (1.24,10.4)	4.14 (1.43,12.0)
Infection severity	Not hospitalised	1	1	1	1
	Hospitalised	0.72 (0.52,1.00)	0.71 (0.51,0.97)	1.21 (0.86,1.70)	1.20 (0.86,1.68)

OR odds ratio; CI confidence interval; SIMD Scottish Index of Multiple Deprivation; COPD chronic obstructive pulmonary disease; CHD coronary heart disease; VOC variant of concern. All statistical tests are two-sided.

Supplementary Table 5. Binary logistic regression models of the factors associated with symptoms at 12 months adjusted for symptoms at six months (unadjusted odds ratios)

		Altered taste N=1,614	Altered smell N=1,794	Confusion N=2,404	Hearing problems N=22,839	Dry cough N=21,653	Cough with phlegm N=21,627
		OR (95% CI)	OR (95% CI)	OR (95% CI)	OR (95% CI)	OR (95% CI)	OR (95% CI)
Covid-19 status	Never infected	1	1	1	1	1	1
	Symptomatic	0.24 (0.16,0.37)	0.20 (0.12,0.33)	0.48 (0.40,0.59)	1.59 (1.38,1.83)	1.39 (1.28,1.53)	1.11 (1.02,1.22)
Age		0.99 (0.99,1.01)	1.00 (0.99,1.01)	1.00 (0.99,1.01)	1.01 (1.00,1.01)	0.99 (0.99,0.99)	0.99 (0.99,0.99)
Sex	Female	1	1	1	1	1	1
	Male	0.99 (0.80,1.24)	1.00 (0.99,1.01)	1.04 (0.87,1.25)	0.84 (0.73,0.97)	0.87 (0.79,0.95)	1.01 (0.92,1.10)
Ethnic group	White	1	1	1	1	1	1
	South Asian	1.51 (0.38,6.07)	0.44 (0.08,2.41)	1.17 (0.58,2.37)	0.18 (0.05,0.74)	0.96 (0.64,1.45)	1.03 (0.69,1.54)
	Black	-	-	2.28 (0.44,11.8)	0.57 (0.14,2.33)	0.59 (0.24,1.47)	0.35 (0.11,1.11)
	Other	1.66 (0.58,4.81)	1.76 (0.71,4.39)	0.74 (0.35,1.55)	0.82 (0.43,1.54)	0.68 (0.44,1.07)	1.01 (0.69,1.48)
	Missing	1.37 (0.74,2.55)	1.87 (1.04,3.35)	1.58 (0.97,2.57)	0.80 (0.52,1.21)	0.71 (0.54,0.94)	0.94 (0.73,1.21)
SIMD quintile	1 (most deprived)	1	1	1	1	1	1
	2	0.87 (0.64,1.16)	0.97 (0.73,1.28)	0.85 (0.68,1.07)	0.91 (0.75,1.11)	1.04 (0.91,1.18)	1.01 (0.88,1.15)
	3	0.81 (0.60,1.10)	0.96 (0.72,1.29)	0.97 (0.76,1.25)	0.77 (0.62,0.94)	0.94 (0.82,1.08)	0.82 (0.71,0.94)
	4	0.98 (0.72,1.32)	1.09 (0.82,1.45)	0.95 (0.75,1.22)	0.77 (0.63,0.95)	0.86 (0.75,0.98)	0.84 (0.73,0.96)
	5 (least deprived)	1.04 (0.76,1.43)	0.99 (0.74,1.33)	1.18 (0.91,1.53)	0.61 (0.49,0.76)	0.74 (0.64,0.85)	0.68 (0.59,0.79)
Pre-existing long-term conditions	0	1	1	1	1	1	1
	1	0.91 (0.68,1.21)	0.89 (0.67,1.19)	1.21 (0.96,1.52)	1.18 (0.96,1.45)	1.47 (1.30,1.66)	1.37 (1.20,1.55)
	2-3	1.17 (0.88,1.55)	0.87 (0.67,1.14)	0.98 (0.80,1.21)	1.99 (1.68,2.35)	1.54 (1.37,1.73)	1.78 (1.59,1.99)
	≥4	0.74 (0.46,1.19)	0.95 (0.60,1.50)	0.91 (0.67,1.22)	3.09 (2.43,3.92)	2.37 (2.00,2.83)	2.31 (1.92,2.77)
Asthma/bronchitis/ COPD	No	1	1	1	1	1	1
	Yes	0.99 (0.79,1.23)	1.05 (0.84,1.30)	0.89 (0.74,1.06)	1.47 (1.27,1.71)	1.38 (1.25,1.53)	1.93 (1.76,2.12)
CHD	No	1	1	1	1	1	1
	Yes	1.03 (0.58,1.82)	1.43 (0.78,2.64)	0.88 (0.58,1.32)	2.03 (1.57,2.64)	1.46 (1.20,1.77)	1.32 (1.08,1.63)
Depression/anxiety	No	1	1	1	1	1	1
	Yes	0.98 (0.81,1.20)	1.04 (0.87,1.26)	0.82 (0.69,0.97)	1.70 (1.48,1.95)	1.37 (1.25,1.49)	1.40 (1.28,1.53)
Diabetes	No	1	1	1	1	1	1
	Yes	0.69 (0.44,1.09)	0.73 (0.47,1.15)	0.96 (0.70,1.32)	1.42 (1.11,1.82)	1.37 (1.16,1.62)	1.33 (1.12,1.58)
Variant period	preVOC	1	1	1	1	1	1
	No dominant (1)	1.35 (1.04,1.77)	1.07 (0.83,1.38)	1.17 (0.96,1.43)	0.90 (0.76,1.07)	0.86 (0.77,0.96)	0.78 (0.70,0.87)
	Alpha	1.37 (0.83,2.27)	1.21 (0.71,2.06)	0.92 (0.66,1.28)	0.80 (0.59,1.08)	0.88 (0.73,1.06)	0.80 (0.66,0.96)
	No dominant (2)	0.86 (0.46,1.60)	0.58 (0.32,1.04)	0.66 (0.36,1.20)	0.79 (0.47,1.32)	1.05 (0.78,1.40)	0.66 (0.47,0.93)
	Delta	0.91 (0.71,1.15)	0.79 (0.63,0.99)	1.16 (0.94,1.45)	1.12 (0.94,1.33)	1.02 (0.90,1.14)	0.79 (0.70,0.89)

	No dominant (3)	2.75 (0.57,13.4)	1.19 (0.33,4.28)	0.97 (0.06,15.6)	1.00 (0.24,4.13)	0.97 (0.38,2.44)	1.07 (0.45,2.51)
Vaccinated at test	No	1	1	1	1	1	1
	1 dose	0.81 (0.53,1.25)	0.69 (0.46,1.05)	0.94 (0.67,1.31)	0.92 (0.67,1.26)	0.95 (0.78,1.16)	0.81 (0.66,1.00)
	≥2 doses	0.76 (0.61,0.95)	0.82 (0.66,1.01)	1.13 (0.91,1.39)	1.25 (1.06,1.47)	1.12 (1.01,1.26)	0.95 (0.85,1.07)
Infection severity	Not hospitalised	1	1	1	1	1	1
	Hospitalised	1.12 (0.66,1.90)	0.83 (0.50,1.38)	0.63 (0.47,0.86)	2.48 (1.82,3.37)	1.24 (0.95,1.63)	1.47 (1.14,1.89)

OR odds ratio; CI confidence interval; SIMD Scottish Index of Multiple Deprivation; COPD chronic obstructive pulmonary disease; CHD coronary heart disease; VOC variant of concern. All statistical tests are two-sided.

Supplementary Table 6. Prevalence of symptoms reported at six- and 12-month follow-up among people who had symptomatic SARS-CoV-2 infection, stratified by trajectory of self-reported recovery status.

	Recovery status		
	Constant N=9,839	Deterioration N=1,497	Improvement N=1,611
	N(%)	N(%)	N(%)
Altered taste - 6 months	1,017 (10.3)	167 (11.2)	269 (16.7)
Altered taste - 12 months	807 (8.20)	200 (13.4)	118 (7.32)
Altered smell - 6 months	1,180 (12.0)	188 (12.6)	311 (19.3)
Altered smell - 12 months	930 (9.45)	235 (15.7)	136 (8.44)
Confusion - 6 months	1,328 (13.5)	217 (14.5)	325 (20.2)
Confusion - 12 months	1,259 (12.8)	304(20.3)	194 (12.0)
Problems hearing - 6 months	540 (5.49)	98 (6.55)	123 (7.64)
Problems hearing - 12 months	630 (6.40)	134 (8.95)	103 (6.39)
Dry cough - 6 months	1,120 (11.4)	193 (12.9)	246 (15.3)
Dry cough - 12 months	1,410 (14.3)	322 (21.5)	214 (13.3)
Productive cough - 6 months	995 (10.1)	196 (13.1)	217 (13.5)
Productive cough - 12 months	1,291 (13.1)	323 (21.6)	205 (12.7)

Supplementary Table 7. Binary logistic regression models of the factors associated with symptoms at 18 months referent to six months

		Altered taste N=435	Altered smell N=498	Confusion N=823	Hearing problems N=5,606	Dry cough N=5,304	Cough with phlegm N=5,329
		OR (95% CI)	OR (95% CI)	OR (95% CI)	OR (95% CI)	OR (95% CI)	OR (95% CI)
Covid-19 status	Never infected	1.00	1.00	1.00	1.00	1.00	1.00
	Symptomatic	0.64 (0.19,2.17)	0.13 (0.02,1.02)	0.63 (0.39,1.00)	1.69 (1.22,2.33)	1.54 (1.24,1.90)	1.43 (1.15,1.77)
Age		0.99 (0.98,1.01)	1.01 (0.99,1.03)	1.01 (0.99,1.02)	1.01 (1.00,1.02)	0.99 (0.99,1.00)	0.99 (0.98,0.99)
Sex	Female	1.00	1.00	1.00	1.00	1.00	1.00
	Male	0.83 (0.51,1.35)	1.14 (0.73,1.79)	0.99 (0.72,1.37)	0.80 (0.60,1.05)	0.79 (0.66,0.95)	1.12 (0.93,1.35)
Ethnic group	White	1.00	1.00	1.00	1.00	1.00	1.00
	South Asian	-	-	1.61 (0.36,7.29)	0.71 (0.17,2.96)	1.03 (0.48,2.19)	0.65 (0.26,1.64)
	Black	-	-	-	1.05 (0.14,8.05)	2.99 (0.94,9.55)	0.90 (0.21,3.98)
	Other	2.61 (0.27,24.9)	1.06 (0.16,7.03)	0.47 (0.13,1.70)	0.73 (0.17,3.04)	1.09 (0.51,2.33)	1.10 (0.53,2.25)
	Missing	4.43 (0.95,20.8)	1.65 (0.47,5.78)	2.63 (0.91,7.54)	0.46 (0.17,1.25)	0.77 (0.46,1.29)	0.52 (0.28,0.96)
SIMD quintile	1 (most deprived)	1.00	1.00	1.00	1.00	1.00	1.00
	2	0.93 (0.51,1.68)	1.21 (0.69,2.12)	0.91 (0.61,1.36)	0.87 (0.61,1.24)	1.09 (0.84,1.40)	1.06 (0.82,1.36)
	3	1.52 (0.83,2.80)	1.48 (0.83,2.63)	1.14 (0.74,1.75)	0.91 (0.64,1.31)	0.96 (0.73,1.25)	0.82 (0.63,1.08)
	4	1.49 (0.82,2.72)	1.27 (0.72,2.23)	1.21 (0.78,1.89)	0.58 (0.38,0.88)	1.05 (0.81,1.37)	0.86 (0.66,1.13)
	5 (least deprived)	2.31 (1.14,4.67)	3.04 (1.57,5.89)	1.86 (1.14,3.04)	0.69 (0.46,1.02)	1.02 (0.78,1.34)	0.75 (0.56,1.00)
Pre-existing long-term conditions	0	1.00	1.00	1.00	1.00	1.00	1.00
	1	1.17 (0.58,2.38)	1.16 (0.61,2.21)	0.81 (0.52,1.27)	1.17 (0.80,1.73)	1.39 (1.08,1.77)	1.35 (1.04,1.75)
	2-3	1.28 (0.68,2.41)	1.40 (0.78,2.51)	0.83 (0.56,1.24)	1.82 (1.30,2.55)	1.38 (1.07,1.77)	1.51 (1.17,1.95)
	≥4	2.18 (0.57,8.37)	2.11 (0.48,9.32	1.43 (0.74,2.77)	2.04 (1.18,3.54)	1.29 (0.82,2.02)	2.62 (1.74,3.94)
Asthma/bronchitis/ COPD	No	1.00	1.00	1.00	1.00	1.00	1.00
	Yes	0.82 (0.48,1.40)	1.18 (0.71,1.98)	0.86 (0.61,1.21)	1.19 (0.89,1.59)	1.24 (1.01,1.52)	1.29 (1.05,1.59)
CHD	No	1.00	1.00	1.00	1.00	1.00	1.00
	Yes	2.81 (0.91,8.69)	1.04 (0.31,3.42)	0.58 (0.27,1.24)	1.11 (0.65,1.90)	1.31 (0.87,1.96)	1.16 (0.77,1.75)
Depression/anxiety	No	1.00	1.00	1.00	1.00	1.00	1.00
	Yes	1.09 (0.72,1.66)	0.99 (0.67,1.47)	0.86 (0.63,1.18)	1.35 (1.03,1.76)	1.19 (0.99,1.43)	1.09 (0.90,1.32)
Diabetes	No	1.00	1.00	1.00	1.00	1.00	1.00
	Yes	0.57 (0.20,1.64)	1.92 (0.57,6.43)	0.83 (0.42,1.62)	0.84 (0.51,1.38)	1.23 (0.86,1.77)	0.90 (0.62,1.30)
Variant period	preVOC	1.00	1.00	1.00	1.00	1.00	1.00
	No dominant (1)	0.74 (0.47,1.15)	0.90 (0.60,1.36)	0.85 (0.62,1.17)	0.96 (0.73,1.27)	1.02 (0.84,1.23)	0.79 (0.65,0.96)
	Alpha	0.59 (0.28,1.23)	0.51 (0.25,1.04)	0.77 (0.47,1.26)	0.95 (0.60,1.49)	1.06 (0.79,1.44)	0.89 (0.65,1.22)

	No dominant (2)	0.44 (0.13,1.46)	0.73 (0.24,2.22)	0.79 (0.31,2.04)	1.21 (0.49,3.00)	1.09 (0.60,1.97)	1.27 (0.71,2.26)
Vaccinated	No	1.00	1.00	1.00	1.00	1.00	1.00
	1 dose	3.23 (0.83,13.4)	1.77 (0.44,7.08)	1.04 (0.46,2.32)	0.77 (0.38,1.57)	0.81 (0.50,1.34)	0.75 (0.44,1.28)
	≥2 doses	Omitted	Omitted	Omitted	0.51 (0.06,4.26)	1.27 (0.40,3.98)	1.41 (0.47,4.24)
Infection severity	Not hospitalised	1.00	1.00	1.00	1.00	1.00	1.00
	Hospitalised	1.02 (0.42,2.49)	0.83 (0.35,1.97)	0.80 (0.50,1.26)	1.77 (1.15,2.73)	1.20 (0.84,1.72)	1.20 (0.83,1.75)

OR odds ratio; CI confidence interval; SIMD Scottish Index of Multiple Deprivation; COPD chronic obstructive pulmonary disease; CHD coronary heart disease; VOC variant of concern. All statistical tests are two-sided.
Odds ratios are adjusted.

Supplementary Table 8. Binary logistic regression models of the factors associated with symptoms at 18 months referent to six months (unadjusted odds ratios)

		Altered taste N=435	Altered smell N=498	Confusion N=823	Hearing problems N=5,606	Dry cough N=5,304	Cough with phlegm N=5,329
		OR (95% CI)	OR (95% CI)	OR (95% CI)	OR (95% CI)	OR (95% CI)	OR (95% CI)
Covid-19 status	Never infected	1	1	1	1	1	1
	Symptomatic	0.51 (0.16,1.60)	0.09 (0.01,0.70)	0.66 (0.43,1.00)	1.59 (1.18,2.14)	1.55 (1.27,1.88)	1.30 (1.07,1.59)
Age		0.99 (0.98,1.01)	1.01 (1.00,1.03)	1.00 (0.99,1.01)	1.01 (1.00,1.02)	0.99 (0.99,1.00)	0.99 (0.99,1.00)
Sex	Female	1	1	1	1	1	1
	Male	0.95 (0.60,1.51)	1.21 (0.80,1.84)	1.03 (0.76,1.38)	0.76 (0.58,0.98)	0.75 (0.63,0.89)	1.02 (0.85,1.22)
Ethnic group	White	1	1	1	1	1	1
	South Asian	-	-	1.54 (0.37,6.51)	0.59 (0.14,2.43)	1.10 (0.52,2.33)	0.68 (0.27,1.71)
	Black	-	-	-	1.07 (0.14,8.04)	2.71 (0.86,8.53)	0.95 (0.22,4.11)
	Other	2.69 (0.30,24.3)	0.99 (0.16,5.97)	0.53 (0.15,1.82)	0.59 (0.14,2.43)	1.08 (0.51,2.29)	1.25 (0.62,2.54)
	Missing	4.03 (0.89,18.2)	1.65 (0.51,5.33)	2.59 (0.93,7.27)	0.42 (0.15,1.13)	0.74 (0.45,1.23)	0.51 (0.28,0.94)
SIMD quintile	1 (most deprived)	1	1	1	1	1	1
	2	0.97 (0.55,1.70)	1.24 (0.73,2.12)	0.91 (0.62,1.35)	0.86 (0.61,1.21)	1.04 (0.81,1.34)	1.01 (0.79,1.30)
	3	1.43 (0.79,2.58)	1.44 (0.83,2.49)	1.10 (0.72,1.67)	0.84 (0.59,1.20)	0.88 (0.67,1.14)	0.77 (0.59,1.01)
	4	1.42 (0.80,2.53)	1.18 (0.69,2.03)	1.23 (0.80,1.88)	0.52 (0.34,0.78)	0.94 (0.73,1.22)	0.78 (0.60,1.02)
	5 (least deprived)	2.21 (1.12,4.38)	2.83 (1.50,5.34)	1.89 (1.18,3.02)	0.59 (0.40,0.87)	0.88 (0.68,1.15)	0.65 (0.49,0.86)
Pre-existing long-term conditions	0	1	1	1	1	1	1
	1	1.18 (0.63,2.20)	1.43 (0.80,2.55)	0.78 (0.51,1.18)	1.28 (0.89,1.85)	1.46 (1.16,1.85)	1.41 (1.10,1.80)
	2-3	1.16 (0.69,1.93)	1.62 (0.97,2.70)	0.70 (0.50,0.99)	2.27 (1.70,3.05)	1.52 (1.23,1.89)	1.57 (1.25,1.96)
	≥4	1.64 (0.56,4.78)	3.01 (0.83,10.8)	1.06 (0.62,1.81)	2.87 (1.85,4.47)	1.59 (1.10,2.30)	2.77 (2.00,3.82)
Asthma/bronchitis/ COPD	No	1	1	1	1	1	1
	Yes	0.94 (0.60,1.47)	1.32 (0.85,2.07)	0.79 (0.58,1.07)	1.61 (1.24,2.10)	1.45 (1.20,1.74)	1.59 (1.32,1.92)
CHD	No	1	1	1	1	1	1
	Yes	2.12 (0.76,5.91)	1.72 (0.60,4.91)	0.63 (0.31,1.26)	1.80 (1.11,2.93)	1.42 (0.98,2.06)	1.59 (1.10,2.31)
Depression/anxiety	No	1	1	1	1	1	1
	Yes	1.00 (0.68,1.47)	1.06 (0.74,1.52)	0.82 (0.62,1.08)	1.74 (1.36,2.24)	1.34 (1.13,1.58)	1.27 (1.07,1.51)
Diabetes	No	1	1	1	1	1	1
	Yes	0.78 (0.31,1.91)	2.87 (0.95,8.65)	0.83 (0.46,1.49)	1.45 (0.93,2.26)	1.39 (1.01,1.90)	1.42 (1.03,1.97)
Variant period	preVOC	1	1	1	1	1	1
	No dominant (1)	0.75 (0.49,1.13)	0.87 (0.59,1.28)	0.82 (0.61,1.10)	1.12 (0.86,1.46)	1.11 (0.93,1.33)	0.86 (0.71,1.03)
	Alpha	0.72 (0.37,1.40)	0.50 (0.26,0.95)	0.75 (0.48,1.18)	0.99 (0.65,1.50)	1.08 (0.82,1.43)	0.90 (0.67,1.21)

	No dominant (2)	0.94 (0.35,2.50)	0.78 (0.30,2.02)	0.95 (0.41,2.18)	1.04 (0.48,2.28)	1.14 (0.67,1.93)	1.30 (0.80,2.13)
Vaccinated at test	No	1	1	1	1	1	1
	1 dose	2.16 (0.69,6.75)	1.49 (0.45,4.90)	0.97 (0.48,1.95)	0.97 (0.52,1.79)	0.93 (0.60,1.45)	0.87 (0.54,1.39)
	≥2 doses	-	-	-	0.73 (0.10,5.41)	1.30 (0.45,3.77)	1.87 (0.70,4.95)
Infection severity	Not hospitalised	1	1	1	1	1	1
	Hospitalised	0.96 (0.42,2.19)	1.04 (0.46,2.35)	0.76 (0.50,1.17)	2.52 (1.68,3.79)	1.44 (1.02,2.02)	1.48 (1.03,2.11)

OR odds ratio; CI confidence interval; SIMD Scottish Index of Multiple Deprivation; COPD chronic obstructive pulmonary disease; CHD coronary heart disease; VOC variant of concern. All statistical tests are two-sided.
