# Supplementary material for: Natural history of long-COVID in a nationwide, population cohort study
Source: Nat Commun. 2023 Jun 13;14:3504. doi: 10.1038/s41467-023-39193-y (PMC10263377; doi:10.1038/s41467-023-39193-y)
Supplement: Supplementary file 3 — Reporting Summary [file 41467_2023_39193_MOESM3_ESM.pdf]

nature portfolio

Corresponding author(s): Jill P Pell
Last updated by author(s): May 15, 2023

Reporting Summary

Nature Portfolio wishes to improve the reproducibility of the work that we publish. This form provides structure for consistency and transparency in reporting. For further information on Nature Portfolio policies, see our Editorial Policies and the Editorial Policy Checklist.

Statistics

For all statistical analyses, confirm that the following items are present in the figure legend, table legend, main text, or Methods section.

n/a	Confirmed	
☐	☒	The exact sample size (*n*) for each experimental group/condition, given as a discrete number and unit of measurement
☐	☒	A statement on whether measurements were taken from distinct samples or whether the same sample was measured repeatedly
☐	☒	The statistical test(s) used AND whether they are one- or two-sided<br>*Only common tests should be described solely by name; describe more complex techniques in the Methods section.*
☐	☒	A description of all covariates tested
☐	☒	A description of any assumptions or corrections, such as tests of normality and adjustment for multiple comparisons
☐	☒	A full description of the statistical parameters including central tendency (e.g. means) or other basic estimates (e.g. regression coefficient) AND variation (e.g. standard deviation) or associated estimates of uncertainty (e.g. confidence intervals)
☐	☒	For null hypothesis testing, the test statistic (e.g. *F*, *t*, *r*) with confidence intervals, effect sizes, degrees of freedom and *P* value noted<br>*Give P values as exact values whenever suitable.*
☒	☐	For Bayesian analysis, information on the choice of priors and Markov chain Monte Carlo settings
☒	☐	For hierarchical and complex designs, identification of the appropriate level for tests and full reporting of outcomes
☒	☐	Estimates of effect sizes (e.g. Cohen's *d*, Pearson's *r*), indicating how they were calculated

Our web collection on statistics for biologists contains articles on many of the points above.

Software and code

Policy information about availability of computer code

Data collection	No software used.
Data analysis	Stata version 16.0

For manuscripts utilizing custom algorithms or software that are central to the research but not yet described in published literature, software must be made available to editors and reviewers. We strongly encourage code deposition in a community repository (e.g. GitHub). See the Nature Portfolio guidelines for submitting code & software for further information.

Data

Policy information about availability of data

All manuscripts must include a data availability statement. This statement should provide the following information, where applicable:

- Accession codes, unique identifiers, or web links for publicly available datasets
- A description of any restrictions on data availability
- For clinical datasets or third party data, please ensure that the statement adheres to our policy

The datasets analysed during the current study are available in the National Services Scotland National Safe Haven, https://www.isdscotland.org/Products-and-Services/eDRIS/Use-of-the-National-Safe-Haven/. This protects the confidentiality of the data and ensures that Information Governance is robust. Applications to access health data in Scotland are submitted to the NHS Scotland Public Benefit and Privacy Panel for Health and Social Care. Information can be found at https://www.informationgovernance.scot.nhs.uk/pbppphsc/

Human research participants

Policy information about studies involving human research participants and Sex and Gender in Research.

Reporting on sex and gender	Of the 23,973 people who completed the questionnaire at both 6 and 12 month follow-up, 14,838 (62%) were female and 9,135 (38%) male.
Population characteristics	See below.
Recruitment	Every adult (>16 years) in Scotland with a positive PCR test from April 2020 was invited by automated SMS text message, along with a comparison group who had had a negative test but never a positive test matched by age, sex, deprivation quintile, and time period of test. Participation was voluntary thus there may be some unmeasurable self-selection bias.
Ethics oversight	Study approval was obtained from the West of Scotland Research Ethics Committee (ref.170 21/WS/0020) and the Public Benefit and Privacy Panel (ref. 2021-0180).

Note that full information on the approval of the study protocol must also be provided in the manuscript.

Field-specific reporting

Please select the one below that is the best fit for your research. If you are not sure, read the appropriate sections before making your selection.

☐ Life sciences ☒ Behavioural & social sciences ☐ Ecological, evolutionary & environmental sciences

For a reference copy of the document with all sections, see nature.com/documents/nr-reporting-summary-flat.pdf

Behavioural & social sciences study design

All studies must disclose on these points even when the disclosure is negative.

Study description	The Long-COVID in Scotland Study (Long-CISS) is an ambidirectional, general population cohort. The National Health Service Scotland notification platform for SARS-CoV-2 PCR results was used to identify eligible participants and invite them via automated SMS text messages.
Research sample	Adults (>16 years) in Scotland with a positive PCR test for SARS-CoV-2 from April 2020 to May 2022 was invited to participate along with a comparison group who had had a negative test but never had a positive test, matched 3:1 by age, sex, and area-based socioeconomic deprivation quintile. Overall response rate was 9%. For these analyses only people who completed a six-month follow-up questionnaire plus at least one subsequent questionnaire (12-month, 18-month or both) were included. Participants with previous symptomatic infection were compared with those never infected. Of the 23,973 participants with both six and 12 month follow-up, 14,838 (62%) were female and median age was 52 years (IQR 38-60).
Sampling strategy	There was no predetermined sample size and no sampling procedure. This is a nationwide study that invited all adults in Scotland who had a positive PCR test for SARS-CoV-2 from April 2020 to May 2022 along with a comparison group.
Data collection	A self-completed online questionnaire collected information on pre-existing health conditions at the time of the index test (first positive test or, for comparison group, most recent negative test) as well as current symptoms, limitations in daily activities and quality of life. Those who had tested positive also provided information on symptoms during the initial infection and current recovery status. Questionnaires were completed 6, 12, and 18 months after the index test. Additional data were obtained through linkage to electronic health records both five years prior their index test and subsequent to the test (up to January 2022) on hospitalizations (Scottish Morbidity Record 01/04), dispensed prescriptions (Prescribing Information System), vaccinations, and death certificates (General Registrar Office). Researchers were not blinded to exposure group or study hypothesis, but nor were they involved in data collection; questionnaires were self-completed and linked data are routinely collected administrative data.
Timing	Questionnaire responses were collected between May 2021 and November 2022.
Data exclusions	53,530 participants were excluded because they reported a previous positive test that was not recorded on the database, 5,687 because they had asymptomatic infections, and 37,343 because they were recruited beyond six months follow-up.
Non-participation	Response rate 9%.
Randomization	This is an observational study with group membership determined by Covid test result. Potential confounders were included in multivariate analysis.

Reporting for specific materials, systems and methods

We require information from authors about some types of materials, experimental systems and methods used in many studies. Here, indicate whether each material, system or method listed is relevant to your study. If you are not sure if a list item applies to your research, read the appropriate section before selecting a response.

Materials & experimental systems

n/a	Involved in the study
☒	☐ Antibodies
☒	☐ Eukaryotic cell lines
☒	☐ Palaeontology and archaeology
☒	☐ Animals and other organisms
☒	☐ Clinical data
☒	☐ Dual use research of concern

Methods

n/a	Involved in the study
☒	☐ ChIP-seq
☒	☐ Flow cytometry
☒	☐ MRI-based neuroimaging
